# A broadband integrated time series (BITS) for longitudinal analyses of the digital divide

**DOI:** 10.1371/journal.pone.0250732

**Published:** 2021-05-26

**Authors:** Elizabeth A. Mack, Edward Helderop, Kangjian Ma, Tony H. Grubesic, John Mann, Scott Loveridge, Ross Maciejewski

**Affiliations:** 1 Department of Geography, The Environment and Spatial Sciences, Michigan State University, East Lansing, Michigan, United States of America; 2 School of Information, University of Texas, Austin, Texas, United States of America; 3 School of Computing, Informatics & Decision Systems Engineering, Arizona State University, Tempe, Arizona, United States of America; 4 Department of Agricultural, Food, and Resource Economics, Michigan State University, East Lansing, Michigan, United States of America; University of Missouri Columbia, UNITED STATES

## Abstract

To evaluate actions taken to implement the Telecommunications Act of 1996, the primary goal of which was to foster competition in the industry, the FCC created a standardized form (Form 477) to collect information about broadband deployment and competition in local telephone service. These data represent the best publicly available record of broadband provision in the United States. Despite the potential benefits offered by this database, there are several nuances to these data related to shifting geographies and reporting requirements that uncorrected, prevent them from being used as an uninterrupted time series for longitudinal analyses. Given the analytical challenges associated with the FCC Form 477 data, the purpose of this paper is to present a solution to the fragmented nature of these data which prevents meaningful longitudinal analyses of the digital divide. Specifically, this paper develops and describes a procedure for producing an integrated broadband time series (BITS) for the last decade (2008–2018). This includes the procedures for using these data, their value to social and economic analysis, and their underlying limitations. The core contribution of this paper is the creation of data infrastructure for investigating the evolution of the digital divide.

## 1. Introduction

The COVID-19 pandemic continues to highlight the importance of high-speed broadband Internet connections to society for a range of business and day-to-day activities (e.g., teleconferencing, retail, education, banking). Unfortunately, the pandemic is also highlighting that disparities in access are still present for many Americans. For example, some efforts to leverage telehealth options during the pandemic, especially in rural areas, are struggling due to poor broadband access [1]. Recent estimates suggest that nearly 21% of students in urban areas are without at-home broadband, while 25% and 37% lack at-home broadband in suburban and rural areas, respectively [2]. Estimates regarding the costs for closing this gap are between $6 and $11 billion [2]. However, determining where these broadband gaps exist remains challenging [3, 4].

In many ways, these challenges represent a more recent incarnation of the digital divide, which refers to uneven access to information technology (IT). Historically this divide referred to access to computers and dial-up Internet connections [5–8]. Research on this divide has uncovered disparities in access related to demographics, socioeconomic status, and educational attainment [9–14]. More recently, the digital divide has become much more complicated and nuanced. Now, it refers to differences in access based on broadband platforms (e.g., cable, fiber, digital subscriber lines (DSL), access speeds, price, and quality of service, among other things [15].

Since 1999, the Federal Communications Commission (FCC) requires that facilities-based broadband providers file Form 477 twice per year to report provision data and inform policy-makers about this divide. Form 477 collects information about broadband connections (200 Kbps or greater) to end-user locations, along with wired and wireless local telephone services and interconnected voice over internet protocol (VoIP) services throughout the United States [16]. This database is one of the outcomes of the Telecommunications Act of 1996 (1996 Act), which directed the U.S. Federal Communications Commission (FCC) to promote competition in the telecommunications sector to lower prices and improve the quality of service for customers [17].

To evaluate actions taken to implement the 1996 Act, the FCC created a standardized form (Form 477) to collect information about broadband deployment and competition in local telephone service [16]. To this day, the information in the Form 477 data represents the best publicly available record of broadband provision in the United States. Despite the potential benefits offered by this database, there are several nuances to these data related to shifting geographies and reporting requirements that prevent them from being used as an uninterrupted time series for longitudinal analyses [18–20]. These nuances, and the essential nature of broadband Internet connections, highlight the need for data that are available to the public to analyze the evolution of the divide in broadband availability over time.

Given the analytical challenges associated with the FCC Form 477 data, the purpose of this paper is to present a solution to the fragmented nature of these data, which prevents meaningful longitudinal analyses of the digital divide. This paper develops and describes a procedure for producing an integrated broadband time series (BITS) for the last decade (2008–2018). This framework includes the procedures for using these data, their value to social and economic analysis, and their underlying limitations. The core contribution of this paper is twofold. First, the BITS are essential because they provide the basis for longer comparative analyses of relative provision levels and the identification of locales that lag behind others consistently. Second, the methodology is important because it offers a path forward for improving some of the comparability issues with the Form 477 data.

## 2. Materials and methods

To support the longitudinal analysis of broadband accessibility in the United States, we synthesized the Fixed Broadband Deployment Data from the Federal Communications Commission (FCC) Form 477. This database contains counts of providers at various geographic scales. We choose to use provider counts as the basis for the BITS based on the precedent set by prior studies which use provider counts in their analyses [21–25]. Furthermore, the number of providers is meaningful in a policy context because broadband policies often endeavor to add providers to a region through subsidies or regulation [22].

We elect to use the Form 477 data instead of other public sources of broadband data, such as the National Broadband Map (NBM), for several reasons [19, 26, 27]. First, the data are distinctly different from the FCC data in that providers were not required to report their information to the National Telecommunications and Information Administration (NTIA). Second, these data contain information for a shorter time period (June 30, 2010—June 30, 2014) than the FCC data. On December 21, 2018 the NBM was formally decommissioned, and responsibilities for broadband mapping were handed over to the FCC [28].

Third, the NBM data also suffer from alternating between 2000 and 2010 Census geographies, which will be discussed in greater detail later in this paper. This divergence in geographies means it is impossible to develop a viable time-series without cross-walking the underlying data. For example, one cannot use the 2010 data, which uses 2000 Census blocks, in combination with the 2011–2014 data, which uses boundaries based on 2010 Census blocks. A further issue with the NBM data related to changing Census geographies is that the switch between 2000 and 2010 tract geographies is inconsistent across states. In particular, the June 2011 version of the NBM is a mix of data using 2000 and 2010 Census geographies. Studies of these data find that only 29 states used 2010 Census geographies [19]. At least 14 states used 2000 Census geographies, and three states (Hawaii, California, and Utah) did not provide information about the Census geography date [19].

For these reasons, we did not consider the NBM data a viable option for constructing the BITS. Instead, we elect to use the Census-based time series collected by the FCC Form 477 database, which offers a more extended period for study (2008-present) than the NBM. Further, unlike the NBM, all facilities-based providers must file Form 477 twice a year, documenting where they (the provider) offer Internet services at speeds exceeding 200 Kbps in at least one direction [16]. These data are available from 1999 onward. However, we start with data reported in 2008 for several reasons. First, the 1999–2007 data are reported at the ZIP code area level and cannot be aggregated to Census-based units, which is the underlying geography for all FCC data from 2008 onward. This mismatch occurs because ZIP codes are not nested neatly within Census blocks, or Census tracts (see [20] for more details).

A second issue with using data before 2008 is related to changes in reporting requirements which affect the number of providers required to report information using Form 477. From 1999 to 2004 only providers with 250 or more high-speed lines were required to report data [29]. This requirement means that information for smaller providers is missing, which creates a discontinuity in the ZIP code provider tabulations. In 2004, the FCC dropped this reporting threshold and required all providers to report data via Form 477 from 2005 onward [30]. However, the FCC does not collect information concerning the number of high-speed lines serviced by a provider in Form 477, which means it is *not* possible to count only providers that meet the high-speed threshold from previous years. As a result, the count of providers in 2005 could be higher than 2004 because of this change in reporting requirements alone.

### 2.1 Broadband data

Due to these issues, the integrated time series starts with the Form 477 data reported as of December 2008, which is the first release of these data using Census-tract geographies. The integration process begins with data collected from the Federal Communications Commission Form 477 database for each of the following three times series: a) 2008–2010, b) 2011–2013, and c) 2014–2018. For each of these periods, we use the most recent release of the data. All had a December timestamp (Table 1) [31]. From 2014 onward, we use the latest version of the data, as indicated by the version number [32].

**Table 1:** Versions of data used.

Year	Description
2008	As of December 31, 2008
2009	As of December 31, 2009
2010	As of December 31, 2010
2011	As of December 31, 2011
2012	As of December 31, 2012
2013	As of December 31, 2013
2014	December 14 version 3
2015	December 15 version 4
2016	December 16 version 2
2017	December 17 version 3
2018	December 18 version 3

The challenge in creating a time series for these data is twofold. First, Form 477 data before 2011 are reported using 2000 Census tract geographies, while data from 2011 onward are reported using 2010 Census tract geographies. This difference in reporting is notable because Census geographies change over time based on population and household dynamics [33]. As a result, it is important to cross-walk the data between 2010 and 2011 to ensure consistency and comparability between 2000 and 2010 Census tract boundaries.

Second, the way that one counts entities across the time series requires standardization. Table 2 documents the data fields for each iteration of the Form 477 data. Of note is that the fields are consistent between 2008 and 2013. Starting in 2014, however, the data fields change and begin to include detailed information about individual providers, such as their FCC registration number, their "doing business as" (DBA) names, holding company name, and holding company number. This holding company information allows one to identify every provider offering service from 2014 onward. This detailed information about holding companies also represents a marked change from the 2008–2013 data, which report only the total number of broadband providers in a Census tract, making it impossible to determine the individual entities offering service.

**Table 2:** Data fields for each iteration of the form 477 data.

2008–2010 Data		2011–2013 Data		2014–2018 Data	
**tract_fips**	Complete Tract FIPS / ANSI Code	**tract_fips**	Complete Tract FIPS / ANSI Code	**LogRecNo**	A logical record number created to relate the broadband deployment tables to the Imputations Table
**state**	State FIPS / ANSI Code	**state**	State FIPS / ANSI Code	**Provider_Id**	filing number (assigned by FCC)
**county**	County FIPS / ANSI Code	**county**	County FIPS / ANSI Code	**FRN**	FCC registration number
**tract**	Tract FIPS / ANSI Code (2 implied decimal places)	**tract**	Tract Code (2 implied decimal places)	**ProviderName**	Provider name
**statename**	State Name	**statename**	State Name	**DBAName**	"Doing business as" name
**countyname**	County Name	**countyname**	County Name	**HoldingCompanyName**	Holding company name (as filed on Form 477)
**tractname**	Tract Name	**tractname**	Tract Name	**HocoNum**	Holding company number (assigned by FCC)
**rfhsc_per_1000_hhs**	Residential Fixed High-Speed Connections per 1000 Households	**rfc_per_1000_hhs**	Residential Fixed High-Speed Connections over 200 kbps in at least one direction per 1000 households	**HocoFinal**	Holding company name (attribution by FCC)
**rfhsc_per_1000_hhs_btop**	Residential Fixed High-Speed Connections per 1000 Households (BTOP/BIP Definition)	**rfc_per_1000_hhs_nbp**	Residential Fixed High-Speed Connections at least 3 Mbps downstream and at least 768 kbps upstream per 1000 Households	**StateAbbr**	2-letter state abbreviation used by the US Postal Service
**total_prov**	Providers of Fixed High-Speed Connections; "1" denotes 1 to 3 providers.	**total_prov**	Providers of Fixed High-Speed Connections over 200 kbps in at least one direction	**BlockCode**	15-digit census block code used in the 2010 US Census
**total_residential_prov**	Providers of Residential Fixed High-Speed Connections; "1" denotes 1 to 3 providers.	**total_residential_prov**	Providers of Residential Fixed High-Speed Connections over 200 kbps in at least one direction	**TechCode**	2-digit code indicating the Technology of Transmission used to offer broadband service
**tmw_prov**	Providers of Mobile High-Speed Connections; "1" denotes 1 to 3 providers.	**total_residential_prov_nbp**	Providers of Residential Fixed Connections at least 3 Mbps downstream and at least 768 kbps upstream	**Consumer**	(0/1) where 1 = Provider can or does offer consumer/mass market/residential service in the block
		tmw_prov*	Providers of Mobile High-Speed Connections over 200 kbps in at least one direction	**MaxAdDown**	Maximum advertised downstream speed/bandwidth offered by the provider in the block for Consumer service
				**MaxAdUp**	Maximum advertised upstream speed/bandwidth offered by the provider in the block for Consumer service
				**Business**	(0/1) where 1 = Provider can or does offer business/government service in the block
				**MaxCIRDown**	Maximum contractual downstream bandwidth offered by the provider in the block for Business service (filer directed to report 0 if the contracted service is sold on a "best efforts" basis without a guaranteed data-throughput rate)
				**MaxCIRUp**	Maximum contractual upstream bandwidth offered by the provider in the block for Business service (filer directed to report 0 if the contracted service is sold on a "best efforts" basis without a guaranteed data-throughput rate)

Additional information reported from 2014 onward includes the transmission technology (e.g., cable, fiber, digital subscriber line (DSL), etc.) and maximum advertised speeds and contractual downstream/upstream speeds for consumers and businesses. The emphasis here is on advertised and contractual speeds. These are very different from the actual speeds at which people and businesses access the Internet. For more details, see [34] or [35]. It is also important to note that the advertised speed data pertain only to *residential service* [32].

Aside from the information reported for each provider, another change with the data from 2014 onward is their tie to Census blocks rather than Census tracts. Blocks provide finer geographic detail than do tracts and are the smallest administrative unit used by the Census Bureau—forming constituent components of larger census units (e.g., block groups and census tracts) [36]. In short, blocks are nested in tracts. Therefore, it is possible to aggregate block-based data to the tract level. When aggregating these data to Census tracts, it is crucial to keep in mind how providers report their information to the FCC. The FCC requires that each provider create a new record when there are any variations in the following information: 1) block, 2) DBA name, or 3) technology of transmission [37]. This requirement means there can be multiple records for the same provider in the same Census tract if they offer service using multiple transmission technologies. Thus, it is essential to filter out duplicate information if one is interested in identifying unique providers offering service.

### 2.2 Crosswalk data

After downloading the broadband data, cross-walk data were obtained from the U.S. Census Bureau to convert 2000 Census geographies to 2010 Census geographies [38]. Cross-walk data are beneficial because they provide information about how Census tracts change from one Census to the next and are used to synthesize Census data over time [33, 39].

These changes happen because the Census structures geographies on specific population thresholds. For example, the Census draws tracts to contain about 4,000 people and 1,600 households. However, at a minimum, tracts will contain at least 1,200 people and 480 households, or a maximum of 8,000 people and 3,200 households [40]. These thresholds mean that Census tracts can merge or split over time.

**Fig 1:**
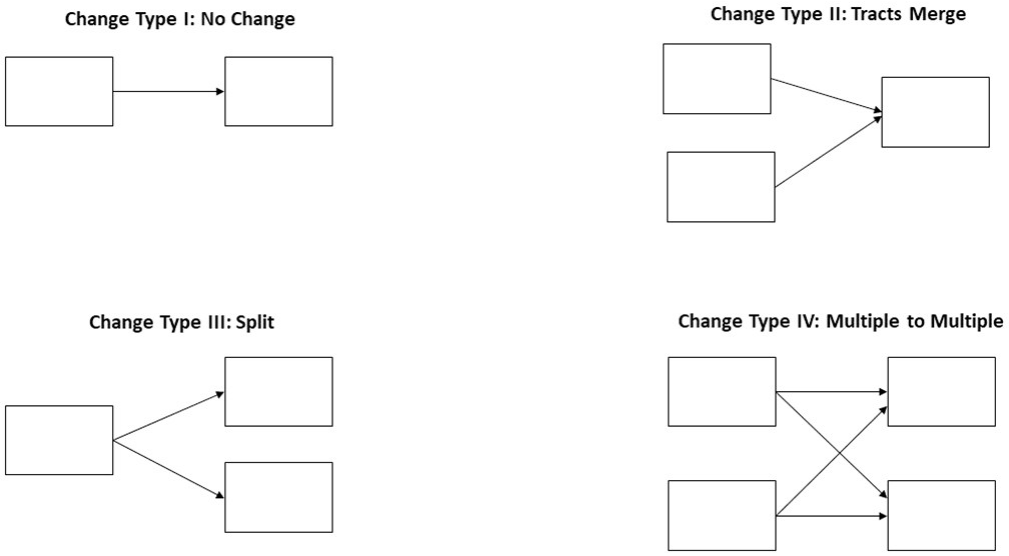
Illustration of change types in the crosswalk data.

Fig 1 outlines four key scenarios of how tracts can change. For this paper, we will refer to these as *change types*. Tracts classified as *Type I* do not change, which means that units display a one-to-one match from the previous Census period and covers the same geographic area. The *Type II* scenario occurs when two or more tracts from 2000 merge into one tract for 2010 because of a loss in either population or housing units. The *Type III* scenario occurs when growth in a 2000 tract requires a split into two or more 2010 tracts. Finally, the *Type IV* scenario occurs when tracts split and are recombined into multiple tracts based on their housing counts. In short, this means that 2000 tracts that split can be recombined into more than one 2010 tract—a key differentiator between Type III and Type IV.

Cross-walk files contain information that detail how 2000 Census tracts relate to 2010 tracts. Specifically, the cross-walk file contains an entry for every 2000 Census tract that contributes any geographic area to a 2010 Census tract, along with a weight value that indicates the specific proportion contribution to the 2010 tract. In our change type classification, an example tract with a change *Type I* would be represented by a single entry in the cross-walk file. This entry would contain the 2000 Census tract ID, the 2010 tract ID to which it contributes, and a weight of 1.0. A change *Type II* for a given 2010 tract would contain multiple entries—one for each of the 2000 Census tracts that contribute to the 2010 tract of interest (recall that change *Type II* means that there must be at least two contributing 2000 tracts). Each of these entries would contain the ID of a 2000 Census tract, the ID of the 2010 tract of interest, and a weight value indicating how much of that 2000 tract’s geographic area contributes to the 2010 tract. This pattern continues for change *Types III* and *IV*, with each contribution between any given pair of 2000 and 2010 tracts represented as one entry in the cross-walk file. There are cross-walks based on population counts, and also housing unit counts. For this study, we use a cross-walk file based on housing units, which mirrors information from a recent report released from the FCC about internet access services [41] and information about rates of return by study area for housing units, land area and density data [42].

### 2.3 Methods

The broadband integrated time series (BITS) dataset produced in this paper is for the contiguous United States, excluding data for Alaska, Hawaii, and U.S territories (American Samoa, Guam, Northern Mariana Islands, Puerto Rico, and Virgin Islands). After removing tracts that did not pertain to the contiguous 48 states and the District of Columbia, we align the 2000 tract geographies for the 2008–2010 time series and the 2011–2013 2010 tract geographies. We use cross-walk data from the Census based on housing units for this purpose [38].

#### 2.3.1 Crosswalking the 2008–2010 data

Since the FCC does not provide any information or guidance about translating data based on 2000 Census geographies to 2010 Census tracts, we consider several strategies for comparison:

*Strategy 1*: The first strategy uses a weighted sum. For a given 2010 Census tract, each of its contributing 2000 Census geographies has its number of broadband providers multiplied by the contribution weight of the 2000 Census tract, as provided in the cross-walk file. These weighted provider numbers are then summed and assigned to the 2010 Census tract of interest.
*Strategy 2*: A second strategy uses the *maximum* weight for the 2000 tract that maps to the 2010 tract. This strategy means that we find the tract with the largest weight for all the 2000 tracts contributing to a 2010 tract and assign its provider count to the 2010 tract. If there is a tie, we assign the tract with the highest count of providers to the 2010 tract.
*Strategy 3*: The third strategy uses the maximum weight for the 2000 tract that maps to the 2010 tract, similar to Strategy 2. However, in the event of a tie, the tract with the *minimum* value of providers is assigned. Readers should consider this a conservative tabulation of broadband provision, while the second strategy provides a more optimistic tabulation of provision.
*Strategy 4*: The fourth strategy utilizes a combination of the above strategies. A critical element of this strategy is using information about the change type of individual census tracts (see Fig 1). This strategy is a more nuanced approach that uses the change types present in the cross-walk file to determine the specific assignment strategy. For 2010 tracts that were assigned a change type of *I* or *II*, we use Strategy 2. For change types *III* or *IV*, we use the weighted sum approach of Strategy 1. As discussed in the error analysis later in this paper, this combination of strategies minimizes deviations between years when converting the data based on 2000 tracts to 2010 tracts.

#### 2.3.2 2014–2018 data

After merging the 2008–2010 time series with the 2011 to 2013 time series, the next step was to compute tract-level information from the block data for 2014–2018. In this phase of the data fusion, we identified a count of unique wireline providers, including terrestrial fixed wireless providers. We remove providers offering satellite or other types of broadband by using the information in the "TechCode" field (e.g., TechCode 60 = satellite, TechCode 0 = other types of broadband). To ensure that we include providers offering service to residential and business locales, we eliminate all observations that did not have a value of 1 in the "Consumer" or "Business" field. This combination of business and residential information is consistent with prior versions of the Form 477 data. In a previous "Report and Order and Further Notice of Proposed Rulemaking" dated March 19, 2008 it is noted that: "We therefore require facilities-based providers of wired, terrestrial fixed wireless, and satellite broadband connections to report the number of connections that they have in service to households and businesses in each of the Census Tracts in which they operate" [43].

The next step to processing these data was to filter out duplicate observations for each census block. As mentioned previously, the FCC asks providers to create a new record when there are variations in the block, DBA name, or technology of transmission [37]. We filter out duplicate observations for the same provider by using the holding company name information for each record ("HocoNum" field) to identify each block’s unique holding companies. This field identifies all commonly owned or commonly controlled entities submitting data to the FCC via Form 477 [29]. This field is used rather than the "Doing Business As Name" because the Form 477 data reports information by holding company [29]. For example, the 1999 data release refers to counts of holding companies in a ZIP code [44].

This nuance to the data is important because using the DBA field rather than the holding company information can produce very different tabulation results. For example, if one were to download version 3 of the 2014 Form 477 data for Missouri and tabulate the number of "doing business as names" associated with a holding company number, they would find that "Windstream Corporation" (holding company number 131413) has nine DBA entities associated with it: PAETEC Communications Inc., McLeodUSA Telecommunications Services LLC, PAETEC Business Services, Cavalier Telephone, Talk America Inc, Windstream Iowa Communications, Inc., Windstream Missouri, Inc., Windstream Norlight, Inc., and Windstream NuVox, Inc. In this case, one might identify nine providers (instead of one) if using DBAs as the basis of tabulating counts instead of holding company information—radically inflating provision counts. Therefore, it is important to use holding company information in tabulating provider counts.

After eliminating duplicate holding companies reporting data for a block, the next step was to extract the Census tract ID from the larger block identification number. To accomplish this, we use the first eleven digits from the 15-digit block number, which contains the information about the corresponding tract id for that block number. Once this was complete, it was possible to tabulate the number of holding companies providing service in a tract. Next, we link tracts to broadband data for 2008–2013. This last step creates a harmonized Census tract level dataset of 71,924 tracts for 2008–2018, based on 2010 Census geographies. This is the number of tracts that have data for all eleven years. One can use the open-source R and Python code associated with this paper to incorporate data for future years as they are released.

## 3. Results

Before highlighting changes in broadband deployment between 2008 and 2018, it is important to discuss sources of uncertainty in the BITS dataset. This uncertainty assessment will focus on two points in the dataset. First, changes in provider counts between 2010 and 2011 may have larger than expected deviations due to cross walking the 2008–2010 data to 2010 Census geographies. Second, changes in provider counts between 2013 and 2014 may be related to how the FCC reports provider counts in the Form 477 database. As mentioned previously, the FCC suppresses counts before 2013 but does not suppress them from 2014 onward.

Concerning the deviations between 2010 and 2011, we quantify uncertainty by creating a difference field. We calculate this field by subtracting the number of providers in 2011 from the number of providers in 2010. Positive deviations mean that the number of providers in a tract is larger in 2011 than in 2010. Negative deviations indicate a larger number of providers in a tract in 2010 than in 2011. We consider three potential ranges of broadband uncertainty worth analyzing. The *first range* corresponds to a change of +/- 5 providers between 2010 and 2011. We use this date range because the FCC suppresses provider counts between 2008 and 2013 [31]. Specifically, if a tract has one, two, or three providers, the exact number is not reported and the FCC assigns the tract a value of 1 [31]. In reality, however, this tract could have between 1 and 3 broadband providers. As one might expect, this can lead to a large jump in provider counts between 2010 and 2013. For example, a tract listed as having "1" provider in 2010 may have six providers in 2011. While this increase of five may seem large, that same tract could have had three providers in 2010 –yielding a net increase of three additional providers for 2011. Conversely, a tract in 2011 may appear to have experienced a decline of five providers. Again, this may represent a smaller decrease if the decline in provision resulted in the number of providers falling within the suppression range of one to three providers.

We evaluate two additional ranges of uncertainty in this paper, both of which are more stringent than the +/-5 deviation. The *second range* of uncertainty pertains to deviations of +/- 4, representing a maximum change of four providers. The *third range* of uncertainty pertains to deviations of +/-3, representing a maximum change of three providers. We create three variables to identify or "flag" tracts outside of these three ranges: "flag5", "flag4", and "flag3". Tracts assigned a value of 1 for this field represent locations that fall outside of the specified range of uncertainty and allows analysts to use only those tracts that fall within their desired level of uncertainty. Here, it is important to note that these year-to-year deviations may be present for any of the years in the data series up to 2014 while the practice of suppressing data was in place. Starting in 2014, with the release of the block data, provider identities are no longer suppressed. For the remainder of this paper, the analysis will focus on tracts within the +/-5 range of uncertainty.

Table 3 compares the four different strategies for constructing this database based on the +/-5 deviation metric. These results suggest that Strategy 4 yields the most robust results because it has the fewest tracts (~2%) outside of the specified range of acceptability. This table also highlights that for Strategy 4, error clusters in change Type III—representing a 2000 Census tract split into multiple 2010 Census tracts due to housing unit growth. In this context, Table 3 suggests that the BITS data will be *least* accurate in rapidly growing parts of the country, particularly in high-growth suburban communities.

**Table 3:** Uncertainty assessment of the BITS.

**Number of Tracts Outside Range -5 to 5**					
	Total Tracts	Change Type 1	Change Type 2	Change Type 3	Change Type 4
Strategy 1	1906	86	308	1,338	174
Strategy 2	2529	86	15	2268	160
Strategy 3	2707	86	184	2268	169
Strategy 4	1613	86	15	1,338	174
**Number of Tracts Outside Range -4 to 4**					
	Total Tracts	Change Type 1	Change Type 2	Change Type 3	Change Type 4
Strategy 1	3,887	348	453	2,730	356
Strategy 2	4064	347	38	3383	296
Strategy 3	4347	347	308	3383	309
Strategy 4	3,471	347	38	2,730	356
**Number of Tracts Outside Range -3 to 3**					
	Total Tracts	Change Type 1	Change Type 2	Change Type 3	Change Type 4
Strategy 1	8,367	1,633	724	5,215	795
Strategy 2	7360	1632	147	5040	541
Strategy 3	7763	1632	531	5040	560
Strategy 4	7,789	1,632	147	5,215	795

**Fig 2:**
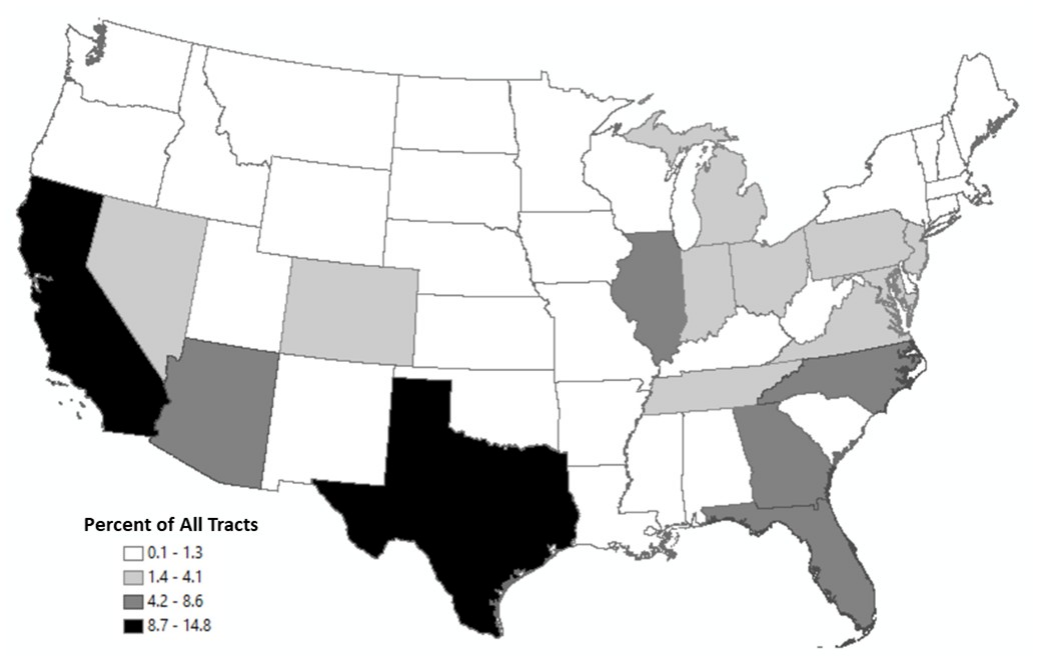
Distribution of tracts outside margin of uncertainty.

To investigate whether this is the case, Table 4 provides a breakdown of tracts outside of the +/- 5 range of uncertainty by their position in the urban-rural hierarchy, as specified by the 2010 rural-urban commuting area (RUCA) codes provided by the United States Department of Agriculture [45]. This table reveals that most of the tracts outside this range of uncertainty (86%) are located in urban areas. Fig 2 highlights that these tracts are primarily located in states with rapidly growing metropolitan areas, including Texas, California, Florida, and Georgia. In Texas, 238 tracts account for just under 15% of all tracts outside the +/-5 deviation metric, followed by California, which has 222 tracts that make up another 14% of these tracts (S1 Appendix).

**Table 4:** Distribution of uncertainty across the urban-rural hierarchy.

	Number of Tracts Outside Uncertainty Range	Percent
1 Metropolitan area core: primary flow within an urbanized area	1,390	86%
2 Metropolitan area high commuting: primary flow 30% or more to an urbanized area	120	7%
3 Metropolitan area low commuting: primary flow 10% to 30% to an urbanized area	12	1%
4 Micropolitan area core: primary flow within an urban cluster of 10,000 to 49,999 (large urban cluster)	50	3%
5 Micropolitan high commuting: primary flow 30% or more to a large urban cluster	10	1%
6 Micropolitan low commuting: primary flow 10% to 30% to a large urban cluster	3	0%
7 Small town core: primary flow within an urban cluster of 2,500 to 9,999 (small urban cluster)	13	1%
8 Small town high commuting: primary flow 30% or more to a small urban cluster	3	0%
9 Small town low commuting: primary flow 10% to 30% to a small urban cluster	2	0%
10 Rural areas: primary flow to a tract outside a urbanized area or urban cluster	10	1%
99: Unclassified	0	0%
**Total Number of Tracts**	**1,613**	

Table 5 presents some information concerning tracts’ local demographic and socioeconomic structure outside of the +/- 5 range. These underlying structures are essential to assess because uncertainty in the BITS for a particular demographic and/or socioeconomic group may limit its utility in some locations and for some populations. This table suggests some minor differences in composition. For example, tracts outside the +/-5 deviation have fewer White and Black residents but more Hispanic and Asian residents. Per capita incomes in tracts with deviations outside the acceptable range are *higher* than tracts inside these ranges. There are also slightly more renters in these same tracts. The differences in income between these locations are particularly salient. These results suggest that tracts with greater deviations have higher incomes than those with lower deviations. This finding is less of a concern than if the reverse were true because tracts with higher incomes are more likely to have higher provision levels than tracts with lower incomes [3, 11].

**Table 5:** Demographic and socio-economic characteristics of uncertain observations.

	Outside +/- 5 Deviation	Within +/-5 Deviation
Percent White*^a^	62%	65%
Percent Black*^a^	10%	13%
Percent Hispanic*^a^	18%	15%
Percent Asian*^a^	7%	4%
Per Capita Income*^b^	$ 32,349	$ 27,105
Percent Owner Occupied*^c^	64%	66%
Percent Renter Occupied*^c^	36%	34%

Note:

*Statistically significant difference as determined by t-test

^a^: 70,619 observations within margin of error, 1,164 observations outside margin of error

^b^: 70,588 observations within margine of error, 1,162 observations outside margin of error

^c^: 70,549 observations within margin of error, 1,161 observations outside margin of error

Concerning the second source of uncertainty between 2013 and 2014, we added a flag variable to the database to identify tracts with a suppressed provider count in 2013 (flag2013). The variable takes on a value of 1 if the number of providers in 2013 is suppressed. This flag is crucial because it indicates observations that may be problematic in 2014 when suppressing provider counts was no longer in practice. To analyze the extent that this change in reporting requirements presents a problem for the database, Table 6 contains a tabulation of the change in provider counts between 2013 and 2014 for tracts that were suppressed in 2013. This table’s logic identifies the extent to which large changes in counts are attributed to this change in reporting requirements. Based on the information detailed in the table, however, this change does not appear to be an issue. 96.8 percent of tracts have a change in provider counts of 5 or less. This change is not unreasonable based on the discussion of provider counts (above). A variation of 3 to 5 providers is dependent on the actual number of providers in the suppressed tract, anywhere between 1 and 3. Only 14 tracts, or 0.2% of all suppressed tracts, have a change in provider counts greater than 7.

**Table 6:** Provider change between 2013 and 2014 for suppressed tracts.

Change 2013–2014	Frequency	Percent	Cumulative Percent
0	160	3.31	3.3
1	1,109	22.91	26.2
2	1,616	33.39	59.6
3	1,057	21.84	81.5
4	531	10.97	92.4
5	211	4.36	96.8
6	105	2.17	99.0
7	37	0.76	99.7
8	9	0.19	99.9
9	5	0.1	100.0

### 3.1 A detailed examination of the BITS

Table 7A–7C provide descriptive statistics for three datasets: 1) the original Form 477 data based on 2000 tracts, 2) the entire BITS data, and 3) the BITS data within the +/- 5 margin of uncertainty. It is important to note that Table 7B and 7C contain more tracts than Table 7A because they are based on 2010 Census geographies, which have more tracts due to population and housing unit growth.

**Table 7:** A comparison of provider counts (2008–2010). **A**. Summary statistics for original form 477 tract data. **B**. Summary statistics for the entire BITS. **C**. Summary statistics for observations within the +/-5 margin of uncertainty.

Variable	Number of Observations	Mean	Std. Deviation	Minimum	Maximum
A					
2008 Provider Count	66,287	6.23	3.29	0	27
2009 Provider Count	66,287	6.43	3.38	0	30
2010 Provider Count	66,287	6.31	3.23	0	31
B					
2008 Provider Count	71,924	5.63	3.11	0	32.23
2009 Provider Count	71,924	5.80	3.20	0	35.40
2010 Provider Count	71,924	5.69	3.08	0	31.51
C					
2008 Provider Count	70,311	5.66	3.09	0	27
2009 Provider Count	70,311	5.83	3.18	0	30
2010 Provider Count	70,311	5.72	3.06	0	31

Table 7B highlights that the entire dataset, without outliers removed, contains slightly higher provider counts than the original FCC data (Table 7A). However, the mean and standard deviation of the entire BITs are slightly lower than the original data. When we remove outliers, the BITS data within the +/-5 margin of uncertainty (Table 7C) closely approximate the original data 2008–2010 data.

To further investigate whether there are unusual changes in the BITS, we conducted two analyses of change over time, one for the entire BITS (2008–2018) and one for the 2014–2018 period, which has no changes in Census geographies and reporting requirements. The logic behind this comparative analysis is to understand the extent that provision changed over an eleven-year period compared to a shorter five-year period. Suppose there are substantial changes in provider counts in the shorter time period. In that case, it follows that changes of a similar magnitude for the longer time period would not be unusual or necessarily related to how we constructed the BITS. Instead, the long-term changes would indicate normal changes in provider counts related to business dynamics in the telecommunications industry. This includes the entry of new providers and the departure of providers related to ongoing mergers and acquisitions within the industry.

**Fig 3:**
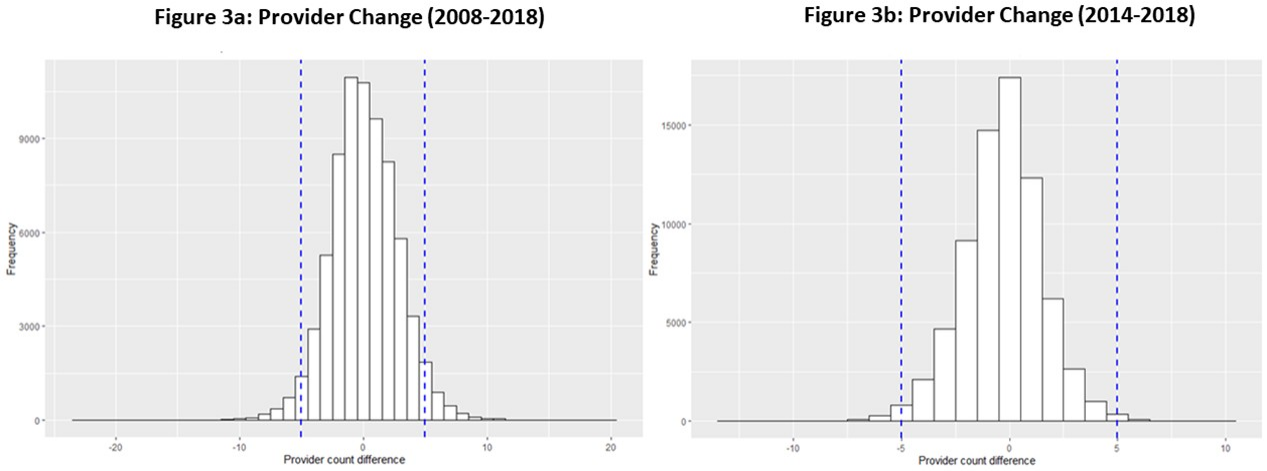
Comparison of provider change over time.

Fig 3 contains histograms of changes in provision between 2008 and 2018 (Fig 3a) and 2014 and 2018 (Fig 3b). The distribution of provider change in the two figures is similar. Fig 3b also reveals that there were large positive and negative changes in provider counts within this shorter period. To complement these figures, S2 Appendix presents information about the full distribution of changes in provider counts. This documentation is particularly important because it helps provide details about the tails of the histograms presented in Fig 3a and 3b. The range of changes between 2008 and 2018 provider counts range from -23 to 20. The range of changes between 2014 and 2018 is between -13 and 10. Where the 2014–2018 period is concerned, analysis reveals that the entry and exit of holding companies within particular areas are driving change. These changes are in line with recent research using data from 2014–2017 which notes "evidence of substantial entry and exit by firms within spatially disaggregated local markets (pg.13)" [46]. This same paper notes that entries are frequently associated with providers of fixed wireless technology [46] which is accounted for by the BITS. An analysis of negative changes indicates that some of these changes reflect consolidation in the telecommunications industry [47, 48]. For example, the acquisition of Windstream by Earthlink in 2016 is visible in these data [48].

That said, the large outliers between 2008 and 2018 (e.g., -23) are related to how the data were cross-walked to convert the 2008–2010 data from 2000 to 2010 tracts. Further analysis of these data reveals clustering in change types 3 and 4. This clustering has to do with the information presented in the cross-walk where one tract has multiple tracts contributing all of their area to a new census tract. In the construction of the BITS, we chose to allocate all providers to the new 2010 tract since it is impossible to tell which providers (or how many providers) offered service in the new tract.

That said, large outliers stemming from this issue *do not* comprise a majority of the dataset. A tabulation of changes in provider counts in the BITS between 2008 and 2018 that exceed the bounds of the changes in provision between 2014 and 2018 (greater than -13 and greater than 10) reveals only 83 observations (0.1%). Users can handle these outliers in several ways. First, readers can use only the observations that fall within the +/-5 margin of uncertainty. Second, readers can remove the observations that exceed the bounds of the changes between 2014–2018, removing these outliers. Third, readers can correct the few observations with large changes from cross-walking the data from 2000 to 2010 Census geographies by electing a different assignment strategy than the one used in this paper.

### 3.2 Longitudinal analyses using the BITS

**Fig 4:**
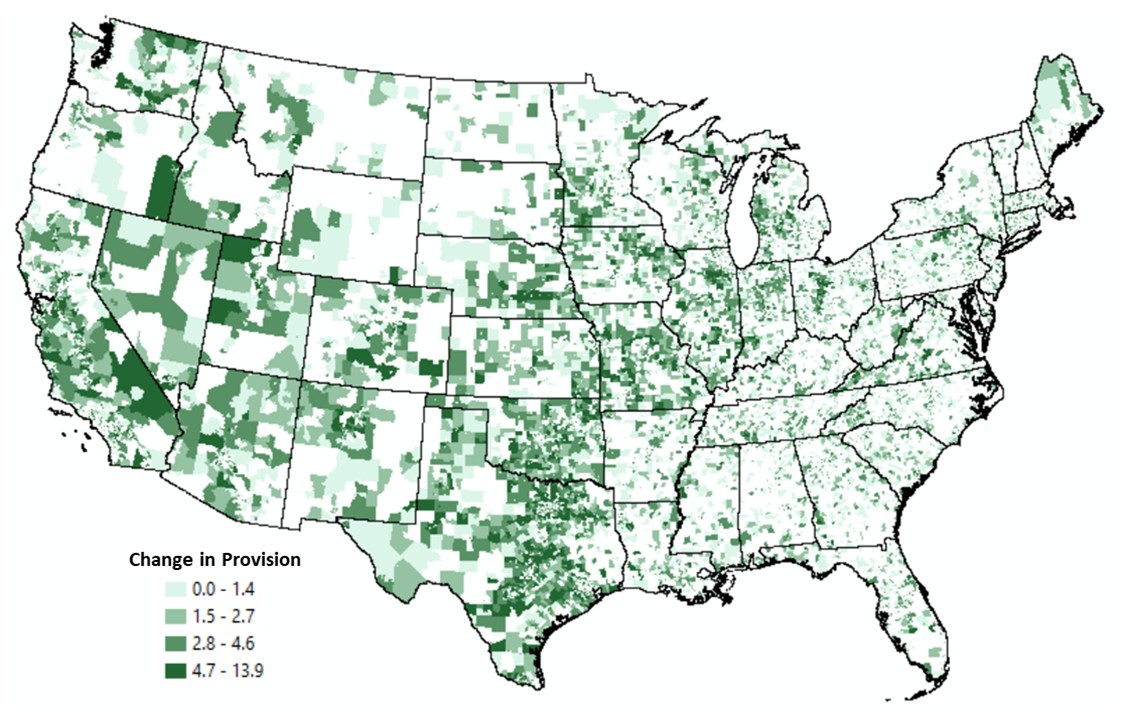
Tracts with positive changes in broadband provision 2008–2018.

**Fig 5:**
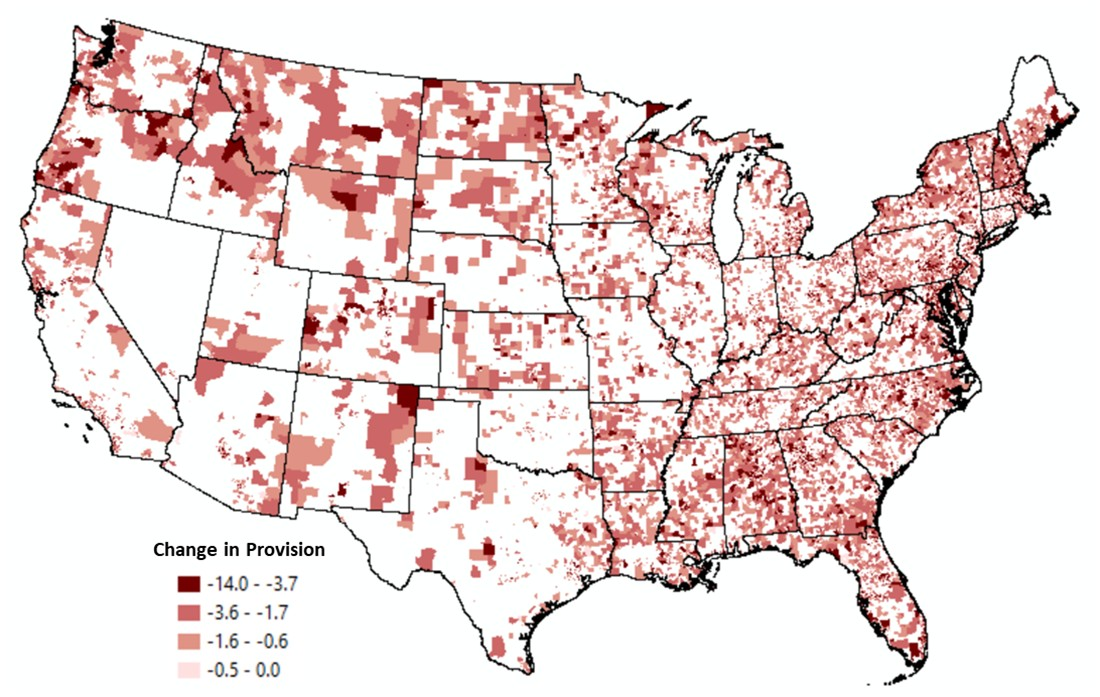
Tracts with negative changes in broadband provision 2008–2018.

Figs 4 and 5 display the change in provision between 2008 and 2018 using only those tracts within the +/- 5 range of uncertainty. There are several notable trends worth highlighting. Fig 4 highlights that many Western states (e.g., Texas, Utah, Nevada, Oklahoma) contain tracts that experienced an increase in provision over these eleven years. This finding, perhaps, reflects population growth in this portion of the country. For example, between 2010 and 2018, the U.S. Census notes that Utah and Texas had the highest population growth in the U.S. [49].

Conversely, Fig 5 displays Census tracts that experienced a decline in provision. These tracts are located in remote areas of Western states, including Wyoming and Montana, as well as tracts located in the Southeast (e.g., Alabama and Mississippi) which contain several geographically isolated, lower-income areas [46] with higher percentages of their populations without broadband service [50]. The Northeast also has several tracts that experienced a decline in provision. This result may reflect maturity and consolidation in the broadband industry.

Table 8 breaks out these changes by computing the ratio of tracts experiencing positive and negative changes in provision by RUCA code to provide some additional clarity on the contextual dynamics of broadband provision during this time. If this ratio exceeds one, more tracts in that RUCA code experienced a positive change in provision than a negative change in provision. It reveals that both urban and rural locations experienced an increase in provision over time. For example, metropolitan areas with reduced commuting levels to the urban core experienced more positive changes in provision. Similar positive gains existed for tracts in both micropolitan and rural areas. This finding may reflect a combination of outward migration from urban centers and initiatives to roll out broadband to underserved areas of the country, including efforts associated with the Connect America Fund [51]. These programs provide funds to telecommunications providers in rural and other unserved areas to help bring both fixed and mobile broadband services to communities at comparable costs in urban areas [52].

**Table 8:** Distribution of provider change across the urban-rural hierarchy.

Rural-Urban Commuting Area (RUCA) Code	Positive Change	Negative Change	No Change	Total	Ratio Positive to Negative Changes
1. Metropolitan area core: primary flow within an urbanized area	21607 (43.1%)	23511 (46.9%)	5047 (10.1%)	50,165	0.92
2. Metropolitan area high commuting: primary flow 30% or more to an urbanized area	3225 (6.4%)	2779 (5.5%)	652 (1.3%)	6,656	1.16
3. Metropolitan area low commuting: primary flow 10% to 30% to an urbanized area	336 (0.7%)	229 (0.5%)	76 (0.2%)	641	1.47
4. Micropolitan area core: primary flow within an urban cluster of 10,000 to 49,999 (large urban cluster)	2054 (4.1%)	1543 (3.1%)	513 (1.0%)	4,110	1.33
5. Micropolitan high commuting: primary flow 30% or more to a large urban cluster	824 (1.6%)	839 (1.7%)	275 (0.5%)	1,938	0.98
6. Micropolitan low commuting: primary flow 10% to 30% to a large urban cluster	185 (0.4%)	170 (0.3%)	53 (0.1%)	408	1.09
7. Small town core: primary flow within an urban cluster of 2,500 to 9,999 (small urban cluster)	916 (1.8%)	906 (1.8%)	303 (0.6%)	2,125	1.01
8. Small town high commuting: primary flow 30% or more to a small urban cluster	344 (0.7%)	355 (0.7%)	117 (0.2%)	816	0.97
9. Small town low commuting: primary flow 10% to 30% to a small urban cluster	153 (0.3%)	135 (0.3%)	50 (0.1%)	338	1.13
10. Rural areas: primary flow to a tract outside an urbanized area or an urban cluster	1486 (3.0%)	1180 (2.4%)	444 (0.9%)	3,110	1.26
99. Not classified	4	0	0	4	N/A
**Total Number of Tracts**	**31,134**	**31,647**	**7,530**	**70,311**	

RUCA codes with a ratio less than one are located in metropolitan cores. This ratio indicates that more tracts in these areas experienced a decline in provision. Again, this could reflect maturity or consolidation in the broadband industry. For example, in 2016, the FCC approved the acquisition of Time Warner Cable and Bright House Networks by Charter Communications [53]. Regardless, this brief snapshot of the data represents only a fraction of the interesting questions that are now possible to pursue and address with the BITS. Many additional applications are also possible.

## 4. Discussion

The COVID-19 pandemic has increased the reliance of people and businesses on high-speed broadband Internet connections. It has also increased the importance of understanding where this essential infrastructure is lacking. The Form 477 database from the Federal Communications Commission (FCC) contains spatial and temporal information about broadband provider counts, which are important and meaningful from a policy perspective because broadband policies often work to add providers to a region, whether that is through subsidies or regulation [22]. Provider counts are also used in broadband-related analyses [21–25]. That said, changes made to the data over time to improve its utility to the research and policy communities make longitudinal analyses difficult, if not nearly impossible, to users without deep knowledge about the evolution of these data. At present, using these data requires extensive knowledge about their intricacies, reporting requirements, and tabulation structures [20, 54]. This paper addresses these issues by designing a methodology for integrating broadband data over time. In doing so, it critically reflected on sources of uncertainty and provided a first look at the spatial patterns of changes in provision using these data.

The BITS are important because they provide the basis for longer comparative analyses of relative provision levels and the identification of locales that lag behind others on a consistent basis. The method is important because it offers a path forward for improving some of the comparability issues with the Form 477 data. The flags and associated confidence metrics allow users to adapt these data to their needs and tolerance for any uncertainty related to data integration. It also provides the opportunity for future research to develop a deeper understanding of differences in provision over time. For example, users may want to analyze the linkage between provision and tract area size using regression analysis. Or, they may wish to include tract area as a control in statistical analyses. The information to accomplish these analyses is available in the BITS.

The creation of this time series also offers the opportunity to exploit variation over time, for example, in econometric panel analyses of broadband and associated health, economic and social impacts and at a finder level of granularity (Census tract-level verus county-level). This coupling enhances our ability to assess historical programs designed to improve broadband access (e.g., the Connect America Fund initiatives) to understand whether relative provision levels have improved over time. This may be especially true where geographic regions, such as tribal land, do not neatly align with county-level boundaries and analysis occurs across multiple states. Longitudinal data are critical for analyses of phenomena that require longer time horizons to ascertain meaningful changes in the condition of people and places over time (e.g., income and job growth, educational attainment, health indicators). From a practical perspective, economic development agencies can use the database to identify areas that are candidates for infrastructure development efforts. One can also use these data to detect changes in broadband markets (positive or negative) over time, indicating changes in communities’ perceived profitability and economic health since decisions about broadband deployment by private providers are made based on profitability assessments [55].

However, when using the BITS, it is important to bear in mind some of the subtleties associated with these data. First, in panel assessments, time fixed effects will be critical to include for modeling variations related to changes in reporting requirements as described in section 2.1. Also, the Form 477 data may overstate the actual coverage of people and businesses [56, 57]. This possibility for overstating coverage is related to several facets of these data. First, in the 2010–2013 time series, providers can report service in a tract if they report just one subscriber in a tract [31]. This reporting nuance does not mean broadband is offered everywhere in the constituent geography. Further, it also means that there are not guarantees of coverage for households within tracts where provision is reported. Information regarding actual household coverage requires a geographic delineation of broadband provision and its coverage from every holding company in the country. This coverage layer would require providers to reveal network connection information—data they consider proprietary.

A second limitation of these data is that they all use the speed threshold of 200 Kbps in at least one direction. While it is possible to ascertain some (albeit limited) information about broadband speeds as of 2011, this is not available in the early part of this time series (e.g. 2008–2010). Between 2011–2013, information about the number of providers of residential fixed connections (at least 3 Mbps downstream, at least 768 kbps upstream) is available. After 2014 higher-resolution information is available about advertised upload and download speeds, but it is not necessarily consistent with the 2011–2013 time series. However, prior work suggests that locales with more providers are likely to have faster broadband speeds [25, 58]. More recent studies find faster wireline speeds in locales with two or more service providers [58]. When combined, these studies suggest that in the absence of speed information in earlier years of the Form 477 data, provider counts may serve as a reasonable proxy for the ability to connect at faster speeds.

The speed aspect of these data does raise an interesting question about what is more important as a measure of broadband availability—is it the basic provision of services as measured by the number of providers? Or, is it the speed of broadband connections (e.g., upload and download)? These are essential questions, but unfortunately, sufficiently high-resolution data to answer this question are not available until 2014. The answer to this question could also depend on the time frame of the study and geographic nuances associated with broadband availability. In the early years of broadband provision, the basic availability of broadband services may have been more important than the service’s speed. More recently, with the rise of participatory Web 2.0 Internet platforms and applications (e.g., social media, cloud computing) post-2004, speed is likely more important. This temporal relationship may or may not be tempered by geographic aspects of the digital divide, as well as the ability to make face-to-face contacts [59]. That said, if users wish to tease out more nuanced aspects of speed from 2014 onward, they may modify the code provided with this paper to begin to evaluate speed-related research questions. The time series over which to do so will be shorter, however.

A third and related limitation of the BITS is that it does not provide information about the platforms (e.g. digital subscriber lines, cable, fiber) for broadband delivery. This limitation stems from the lack of platform information before 2014. That said, studies do note that the likelihood of more platform choice is linked to provider counts [15]. Thus, one can use provider counts as a reasonable proxy for platform choice.

Aside from these nuances to the BITS, there are other considerations about broadband terminology and the construction of the BITS worth noting. The first consideration is related to nuances in broadband terminology since it is crucial to understanding how to interpret both the Form 477 data and the BITS. The provider data is a measure of deployment, meaning that that provider could offer service. The list of providers in a Census tract does not mean that all providers service every household in that tract. Households are likely to have fewer options than listed in that tract. Again, to understand who is served by each provider, a coverage map from each facilities-based provider would need to be submitted to the FCC, as described above. Even if these were available, it would not necessarily mean that households adopted and are using broadband. Studies of un-adopters of broadband certainly highlight that not all people will adopt broadband for cost or use issues, even if available [60]. Blending each of these distinct terms can lead to confusion as to the interpretation of the BITS. For example, it is possible to have a declining number of broadband providers although coverage and broadband adoption are expanding over time. In this example, a declining number of providers could indicate consolidation of the industry via mergers and acquisitions. This result would mean that people formerly serviced by an old provider would be serviced by the new provider. It does not mean that fewer people have service.

Lastly, and perhaps most importantly, the BITS were created based on the authors’ interpretation of the FCC orders about changes in Form 477 reporting requirements. The BITS also represents choices by the authors related to cross-walking the data from 2000 to 2010 Census geographies. As indicated in our discussion of this process, we considered and chose the option that minimized large deviations for years in the dataset that correspond to changes in reporting requirements. These choices are supported by information from FCC orders outlining changes in the Form 477 data [29, 30, 43, 61, 62]. We have also provided the code for creating the BITS to enhance the replicability of this integration process. That said, it is possible to make other choices about data integration. Different choices will produce deviations from the data provided as part of this study.

## 5. Conclusion

As more of our day-to-day activities (e.g., education, commerce, healthcare) move online, it is increasingly important to understand where gaps in digital infrastructure may exist. The current financial climate underscores this need. With shrinking public budgets and a need to pinpoint locations suffering from a chronic shortage of broadband, it is critical for policy-makers to efficiently allocate the human, infrastructural, and policy resources required to improve local conditions. We are hopeful that this paper is a step forward in addressing this need. The paper not only provides a framework for integrating and fusing Form 477 broadband data into a robust time-series database, it provides a user-friendly and harmonized version of the broadband data for use, now. In addition, the BITS includes a suite of open-source code that analysts can modify for their own needs for future analyses and a methodological approach for cross walking Census data, which is important for future analytical efforts that will need to deal with changes in Census geographies. While the BITS is far from perfect, it represents an alternative to the current, shorter time series Form 477 data that are available for evaluating broadband provision and the digital divide.

## Supporting information

S1 Appendix Distribution of uncertainty by state. (DOCX)

S2 Appendix A comparison of changes in broadband provision over time. (DOCX)
