# Supplementary material for: A broadband integrated time series (BITS) for longitudinal analyses of the digital divide
Source: PLoS One. 2021 May 26;16(5):e0250732. doi: 10.1371/journal.pone.0250732 (PMC8153480; doi:10.1371/journal.pone.0250732)
Supplement: S1 Appendix — (DOCX) [file pone.0250732.s001.docx]

**S1 Appendix:** **Distribution of Uncertainty by State**

|  | **Number Tracts with Uncertain Information** | **Percent of All Uncertain Tracts** |
| --- | --- | --- |
| Texas | 238 | 14.8% |
| California | 222 | 13.8% |
| Florida | 139 | 8.6% |
| Georgia | 97 | 6.0% |
| Arizona | 91 | 5.6% |
| North Carolina | 89 | 5.5% |
| Illinois | 84 | 5.2% |
| Colorado | 66 | 4.1% |
| Virginia | 53 | 3.3% |
| Pennsylvania | 49 | 3.0% |
| Ohio | 37 | 2.3% |
| Tennessee | 32 | 2.0% |
| Maryland | 31 | 1.9% |
| Michigan | 31 | 1.9% |
| Nevada | 29 | 1.8% |
| New Jersey | 28 | 1.7% |
| Indiana | 25 | 1.5% |
| Massachusetts | 21 | 1.3% |
| Utah | 20 | 1.2% |
| South Carolina | 19 | 1.2% |
| New York | 17 | 1.1% |
| Wisconsin | 17 | 1.1% |
| Missouri | 16 | 1.0% |
| Alabama | 14 | 0.9% |
| Kentucky | 14 | 0.9% |
| Washington | 14 | 0.9% |
| Minnesota | 13 | 0.8% |
| Oregon | 13 | 0.8% |
| Iowa | 12 | 0.7% |
| Mississippi | 11 | 0.7% |
| New Mexico | 11 | 0.7% |
| Louisiana | 10 | 0.6% |
| South Dakota | 9 | 0.6% |
| Kansas | 8 | 0.5% |
| Idaho | 5 | 0.3% |
| Oklahoma | 5 | 0.3% |
| Montana | 4 | 0.2% |
| Nebraska | 3 | 0.2% |
| West Virginia | 3 | 0.2% |
| Wyoming | 3 | 0.2% |
| Connecticut | 2 | 0.1% |
| Delaware | 2 | 0.1% |
| Arkansas | 1 | 0.1% |
| District of Columbia | 1 | 0.1% |
| Maine | 1 | 0.1% |
| North Dakota | 1 | 0.1% |
| New Hampshire | 1 | 0.1% |
| Vermont | 1 | 0.1% |
| Rhode Island | 0 | 0.0% |
