# Supplementary material for: A broadband integrated time series (BITS) for longitudinal analyses of the digital divide
Source: PLoS One. 2021 May 26;16(5):e0250732. doi: 10.1371/journal.pone.0250732 (PMC8153480; doi:10.1371/journal.pone.0250732)
Supplement: S2 Appendix — (DOCX) [file pone.0250732.s002.docx]

**S2 Appendix: A Comparison of Changes in Broadband Provision Over Time**

| **Change 2014-2018** | **Frequency** |  | **Change 2008-2018** | **Frequency** |
| --- | --- | --- | --- | --- |
| -13 | 1 |  | -23 | 1 |
| -12 | 1 |  | -22 | 0 |
| -11 | 0 |  | -21 | 0 |
| -10 | 0 |  | -20 | 0 |
| -9 | 8 |  | -19 | 0 |
| -8 | 22 |  | -18 | 2 |
| -7 | 87 |  | -17 | 2 |
| -6 | 283 |  | -16 | 0 |
| -5 | 820 |  | -15 | 1 |
| -4 | 2094 |  | -14 | 2 |
| -3 | 4658 |  | -13 | 2 |
| -2 | 9150 |  | -12 | 13 |
| -1 | 14739 |  | -11 | 20 |
| 0 | 17402 |  | -10 | 52 |
| 1 | 12313 |  | -9 | 81 |
| 2 | 6208 |  | -8 | 188 |
| 3 | 2662 |  | -7 | 363 |
| 4 | 984 |  | -6 | 723 |
| 5 | 369 |  | -5 | 1388 |
| 6 | 91 |  | -4 | 2905 |
| 7 | 18 |  | -3 | 5281 |
| 8 | 10 |  | -2 | 8486 |
| 9 | 3 |  | -1 | 10951 |
| 10 | 1 |  | 0 | 10791 |
|  |  |  | 1 | 9629 |
|  |  |  | 2 | 8259 |
|  |  |  | 3 | 5809 |
|  |  |  | 4 | 3312 |
|  |  |  | 5 | 1845 |
|  |  |  | 6 | 893 |
|  |  |  | 7 | 460 |
|  |  |  | 8 | 217 |
|  |  |  | 9 | 111 |
|  |  |  | 10 | 62 |
|  |  |  | 11 | 44 |
|  |  |  | 12 | 10 |
|  |  |  | 13 | 8 |
|  |  |  | 14 | 7 |
|  |  |  | 15 | 1 |
|  |  |  | 16 | 2 |
|  |  |  | 17 | 2 |
|  |  |  | 18 | 0 |
|  |  |  | 19 | 0 |
|  |  |  | 20 | 1 |
